# A New Reflex Sign in Spastic Paraplegia

**Published:** 1918

**Authors:** 


***A New Reflex Sign in Spastic Paraplegia.*** By Dr. ROBERT BING. *Corresp.-Blatt f. Schweizer Aerzte*, April 13, 1918.

Bing calls his new sign the paradoxical ankle reflex. He discovered it first in a case of syphilitic hemiplegia and later established its constant occurrence in organic spastic conditions involving one or both lower extremities.

It is produced in the following way : With the patient on his back, the affected leg is put in the usual position for testing ankle clonus being moderately flexed at hip and knee. The examiner's hand brings the foot into moderate dorsiflexion with consequent slight tension on the Tendo Achilles while a sufficiently heavy percussion hammer is employed to strike the dorsum of the foot at any point along the imaginary line joining the malleoli. In positive cases there results an immediate contraction of the gastrocnemius and a corresponding flexion of the foot at least as marked as is usually obtained for the ordinary ankle—Achilles-jerk. The test is easy to elicit and is of unequivocal significance. It is immaterial whether any particular tendon of the extensor group including the tibialis anticus, extensor proprius hallucis and communis digitorum is struck or not. In negative cases no movement results or else simply a weak extension or dorsiflexion.

This reflex belongs to the group of inverted reflexes where percussion on the extensor side of the limb is followed by contraction of the flexors or vice versa.

The paradoxical and inverted reflex of Piotrowski, in which percussion of the belly of the tibialis anticus produces a smart plantar flexion of the foot. He has found this reflex present in four cases where the other more commonly elicited reflex signs of organic spasticity from cortico-spinal impairment were apparently absent.

